# Modulation of Emotional Category Induced by Temporal Factors in Emotion Recognition

**DOI:** 10.1371/journal.pone.0131636

**Published:** 2015-07-31

**Authors:** Hiroaki Maeshima, Yuichi Yamashita, Tomomi Fujimura, Masato Okada, Kazuo Okanoya

**Affiliations:** 1 Department of Life Sciences, Graduate School of Arts and Sciences, The University of Tokyo, Tokyo, Japan; 2 JST, ERATO, Okanoya Emotional Information Project, Wako, Saitama, Japan; 3 Cognition and Behavior Joint Research Laboratory, RIKEN Brain Science Institute, Wako, Saitama, Japan; 4 Department of Functional Brain Research, National Institute of Neuroscience, National Center of Neurology and Psychiatry, Kodaira, Tokyo, Japan; 5 Human Technology Research Institute, National Institute of Advanced Industrial Science and Technology, Tsukuba, Ibaraki, Japan; 6 Department of Complexity Science and Engineering, Graduate School of Frontier Sciences, The University of Tokyo, Kashiwa, Chiba, Japan; Bournemouth University, UNITED KINGDOM

## Abstract

Categorical perception (CP), the perceptual experience whereby continuous sensory phenomena are perceived as distinct and separate percepts, is one of the most characteristic features of information processing in human cognition. CP is considered as the result of the integration of the top-down processing including background knowledge and verbal labeling and the bottom-up processing such as physical characteristics of the sensory signal. However, the underlying mechanisms governing the integration remain unclear. To address this issue, we focused on the temporal characteristics of CP of facial expression. In the current study, we investigated the contributions of temporal factors in CP processes, using facial expression recognition tasks as an example of CP. Participants completed an identification task and a discrimination task, well-established tasks for evaluating CP of facial expressions, with variable temporal parameters, that is, duration of stimulus presentation and delay time (interval between stimulus and response). The results demonstrated that the emotionally ambiguous stimuli are categorized more distinctively with the extension of delay length, not of stimulus duration. In contrast, the category boundary for facial expressions shifted toward “happy” with extention in stimulus duration, not in delay length. This dissociation between the impact of stimulus duration and delay suggests that there are two processes contributing to CP of facial emotion; one process may reflect the internal processing associated with the length of the delay period including verbal labeling of the stimuli, and the other process may reflect the temporal summation of stimulus inputs, associated with stimulus duration. These findings suggest that paying more attention to temporal factors in CP could be useful for further study of the mechanisms underlying CP.

## Introduction

Categorical Perception (CP) is the perceptual experience whereby continuous sensory phenomena are perceived as distinct and separate percepts. The topic originating in the theory of non-linearity of visual perception [1] is a major topic in cognitive psychology. CP is a ubiquitous feature of information processing in human cognition. For example, continuous changes in colors and vocal sounds are perceived as stereotypical colors [2, 3] and discrete phonemes [4]. Accumulated evidence from the studies of CP suggests that both the top-down processing including background knowledge [5] and “stored exemplar” (that is, memorized sample) [6] and the bottom-up processing such as physical characteristics of the signal are considered to be important for categorization [7, 8]. In other words, CP is the result of the integration of the top-down processing and bottom-up processing [8, 9].

The recognition of facial expressions is a representative example of the perceptual experience of CP [10]. When we observe an emotionally ambiguous facial expression, we perceive it as one of the discrete categories of emotion, such as happiness, sadness, and so on [10, 11, 12, 13, 14].

Higher cognitive functions, such as language, may play a key role in CP of facial expression. For example, CP of faces and colors is attenuated when participants are engaged in an interfering verbal dual task, even though participants are not required to make verbal responses [15, 16, 17]. Similarly, when verbal labeling is prevented, recognition accuracy of stereotypical facial expression is reduced [18]. Another line of research shows that CP of the color perception occurs if the participants’ native language has a word corresponding to a particular color stimulus, that is, within-category discrimination is more difficult than between-category discrimination if the corresponding “color word” exists [3]. These previous studies strongly suggest that higher cognitive functions, including language, play a key role in CP [19]. There exists a study claiming that, based on the assumption that CP is involved in higher cognitive functions such as labeling and symbol manipulations requiring the fine analysis of the stimuli, CP is relatively slow compared to coarse sensory-perceptual processes [20].

In contrast, some researchers have suggested that CP of facial expression occurs during the initial stage of emotion perception. For example, happy or fearful faces can be discriminated at short (<64 ms) presentation durations [21]. In a neuroimaging study, the amygdala response differed as a function of emotional category, even at an initial stage (approximately 150 ms) of facial expression processing [22]. In addition, an electro-encephalogram study revealed that the amplitude [23] and the latency [24] of an early event-related potential component, N170 (observed approximately 170 ms after stimulus onset) change according to emotional category.

In relation to these results, some researchers suggest that facial expression categorization occurs without corresponding words [25], but with neural network model [26]. Moreover, 7-month childs showed CP in facial expressions [27].

These observations suggest that there are two pathways for processing facial expression. This is consistent with the two-process theory in social psychology [28, 29], according to which judgment and decision making are based on two processes: fast, effortless, intuition, and slow, deliberate, reasoning. The two-process theory could be extended to the recognition of facial expression [30], namely, judgments of emotional valence.

The relationship between two pathways and their contribution to CP remain unclear. These questions may be unanswered because little attention has been paid to temporal factors in CP. In most previous CP studies, stimulus presentation duration is fixed and relatively long (>700 ms) [10, 11, 15, 31]. This makes it difficult to investigate the timing of CP. Moreover, the impact of stimulus delay has been investigated in relatively long range (for example, 0 s, 5 s and 10 s), resulting in failing to investigate earlier processing [15].

The aim of the current study is to investigate the contributions of temporal factors in CP processes, using facial expression recognition tasks as an example of CP. To evaluate the degree of CP, an identification task and a discrimination task, well-established tasks for evaluating CP of facial expressions [11], were used. We varied the temporal parameters of these tasks, i.e., duration of stimulus presentation and delay time (interval between stimulus and response). Specifically, stimulus duration and delay are assumed to be related to different perceptual processes. For example, stimulus duration may be related to the accessibility of the stimulus. That is, when a stimulus is presented for a longer duration, participants can shift their attention to multiple stimulus features before making a final judgment [32]. This results in a more consolidated facial recognition judgment [30]. In addition, stimulus duration can be related to the accumulation of stimulus inputs. Light, for example, even with constant intensity, is perceived as stronger when presented for a longer duration [33].

In contrast, changes in delay may be related to the memory system; that is, memory for the stimuli could vary according to the length of the delay. For example, in a shorter delay period, stimuli can be retained as they are (sensory retention [34, 35]); in a longer delay period, memory for stimuli may be modified by higher cognitive processes, such as verbal labeling [36]. In two-pathway theory, longer delay condition corresponds to the slower pathway, in which participants may process the imformation in more detail.

## Material and Methods

### 2.1. Participants

Twenty-one undergraduates and postgraduates (9 men and 12 women, 20–24 years old) participated in this experiment. All participants were native Japanese speakers and right-handed. They had normal or correct-to-normal eyesight, and no history of mental or neurological illness.

### 2.2. Ethics statement

All participants signed an informed consent form prior to participation. The experiments were conducted with the approval of the University of Tokyo Ethics Committee.

### 2.3. Stimuli

**Fig 1:**
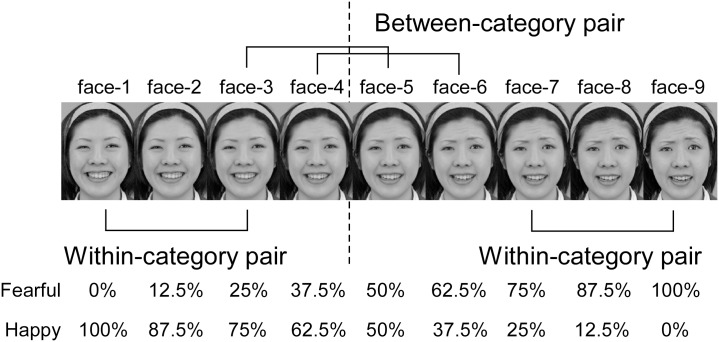
Facial expression continuum used in the experiment. This is a happy-fearful morphing continuum from happy (left endpoint) to fearful (right endpoint). Face pairs that cross the category boundary (dotted line) are referred to as between-category pairs, and face pairs that do not cross the category boundary are referred to as within-category pairs. Morphed images ranged from 100% happy (face-1) to 100% fearful (face-9). Based on the results of the identification task (see Results), the category boundary was determined to be between face-4 and face-5. The actual facial images used in the current experiment were copyrighted, therefore we used the similar images for explanation. These are faces of one of the authors, and there is no copyright infringement. These photos are taken by laboratory of facial information, College of Humanities and Sciences, Nihon University.

To investigate the perception of ambiguous facial expressions, a continuum of facial expressions that changed according to emotional valence was used. Prototypical happy and fearful faces of one male model, selected from Pictures of Facial Affect [37], were used as the endpoints of the facial expression continuum. We created the images morphed in 9 steps from happy to fearful facial expressions in equal physical steps. These faces are the same as used in the previous paper (Fujimura et al., [38]). The continuum of happy-fearful facial expressions included faces that blended the two emotions in the following proportions: face-1 = 100% happiness, 0% fear; face-2 = 87.5% happiness, 12.5% fear; face-3 = 75% happiness, 25% fear; face-4 = 62.5% happiness, 37.5% fear; face-5 = 50% happiness, 50% fear; face-6 = 37.5% happiness, 62.5% fear; face-7 = 25% happiness, 75% fear; face-8 = 12.5% happiness, 87.5% fear; and face-9 = 0% happiness, 100% fear (Fig 1). Each facial stimulus subtended to a visual angle of approximately 6.6° × 4.7°.

### 2.4. Apparatus

Experimental events were controlled by a program written in Presentation (Neurobehavioral Systems). Stimuli were presented on a 21-inch LCD monitor (EIZO Flexscan L887; refresh rate: 60 Hz). The distance between the screen and participants was approximately 80 cm.

### 2.5. Procedure

An identification task and a discrimination task [11], well-established tasks for evaluating CP of facial expressions, were used in the current experiment. To investigate the impact of temporal factors in CP, temporal parameters were manipulated—duration of stimulus presentation and delay time (interval between stimulus and response). There were three stimulus duration conditions: 50 ms, 200 ms, and 750 ms. There were two delay conditions: 200 ms and 900 ms. Therefore, there were six experimental conditions (3 × 2). The experiment was a block design with six conditions. We chose block design to avoid the participants’ having single strategy. That is, when the trials of different conditions are mixed in the same block, participants might solve all trials with a single strategy for the most difficult (short duration/delay) condition. Experimental parameters were fixed during each block and the block of each parameter presented only once. The order of conditions and tasks was pseudo-randomized across participants. The order of faces was randomized across the experiment.

### 2.6. Identification task

**Fig 2:**
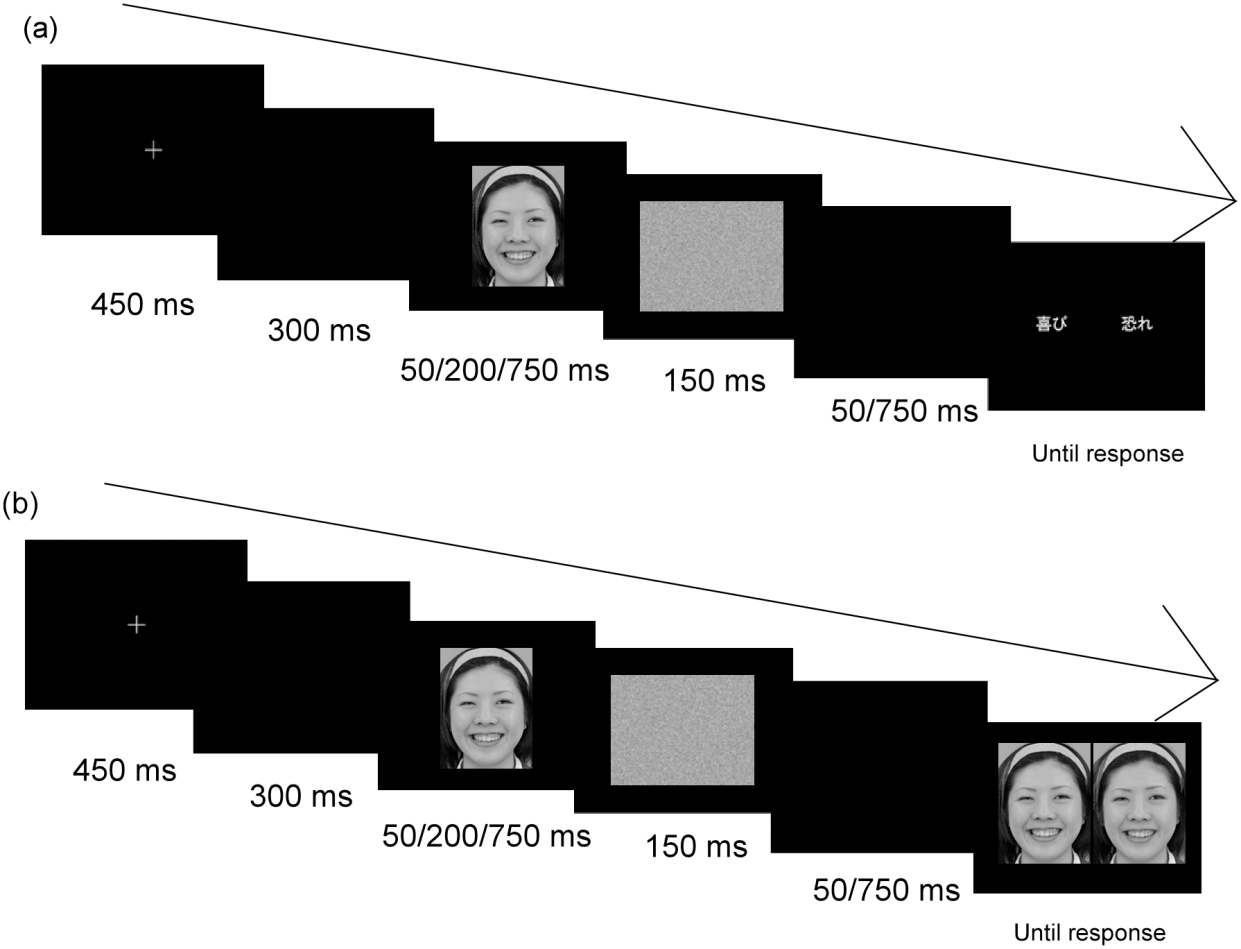
Trial sequence for the (a) identification and (b) discrimination tasks. Each task began with a fixation point (450 ms), followed by a blank screen (300 ms), a target face (50/200/750 ms), a white-noise mask (150 ms), blank screen (50/750 ms), and (a) two emotion words (identification task) or (b) two faces (discrimination task).

In the identification task, participants were required to identify the facial expression of a target stimulus as quickly as possible by choosing between the words “happy” and “fearful.” For example, in a trial, participants may be asked to judge whether the face-2 stimulus (87.5% happy, 12.5% fearful) is happy or fearful by pressing a button. Fig 2a illustrates the procedure for the identification task. Each trial began with a fixation point (450 ms), followed by a blank screen (300 ms), the target face image (50, 200, or 750 ms), and a white noise masking image (150 ms). After a blank interval (50 or 750 ms), two emotion words (happy and fearful) were presented until the participant responded by pressing one of the assigned buttons. The assignment of buttons was right index finger (left button) and right middle finger (right button). Therefore, the period between target face presentation and the two emotion words (participants’ response) was 200 or 900 ms (including masking), which we refer to as the 200 and 900 ms delay conditions, respectively.

There were six blocks of the identification task, one for each of the six different temporal parameter conditions. Each face stimulus was presented eight times per block. Therefore, there were 72 trials in each session. The positions of the two emotion words were counterbalanced across trials.

### 2.7. Discrimination task

In the discrimination task (XAB; [15]), participants were required to discriminate between two faces from the continuum. Fig 2b illustrates the procedure for the discrimination task. Each trial began with a fixation point (450 ms), followed by a blank screen (300 ms), the target face image (X) (50, 200, or 750 ms), and a mask stimulus (150 ms). After a blank interval (50 or 750 ms), two sample faces (A and B) were presented until participants pressed a response button to indicate whether X matched A or B. As in the identification task, the delay period was 200 or 900 ms (including masking). The two sample faces (A and B) were two steps apart on the continuum (e.g., face-1 and face-3). The difference between two faces that were two steps apart corresponded to 25% physical difference. This difference can be considered as appropriate difference to detect CP of facial expression based on the previous study with Japanese participants using the same stimuli (same model and same facial expressions) [39]. The target face was identical to one of the two sample faces. Participants were requested to choose the face that matched the target face by pressing a button. The assignment of buttons was right index finger (left button) and right middle finger (right button). There were four possible presentation types (AAB, BAB, ABA, and BBA) for each of the seven possible face pairs. Each type of face pair was presented twice in each block, resulting in 56 trials per block (4 × 2 × 7). Similar to the identification task, there were six blocks for the discrimination task, one for each of the six different conditions. The order and the position of faces were randomized in each block.

## Results

### 3.1. Identification task

**Fig 3:**
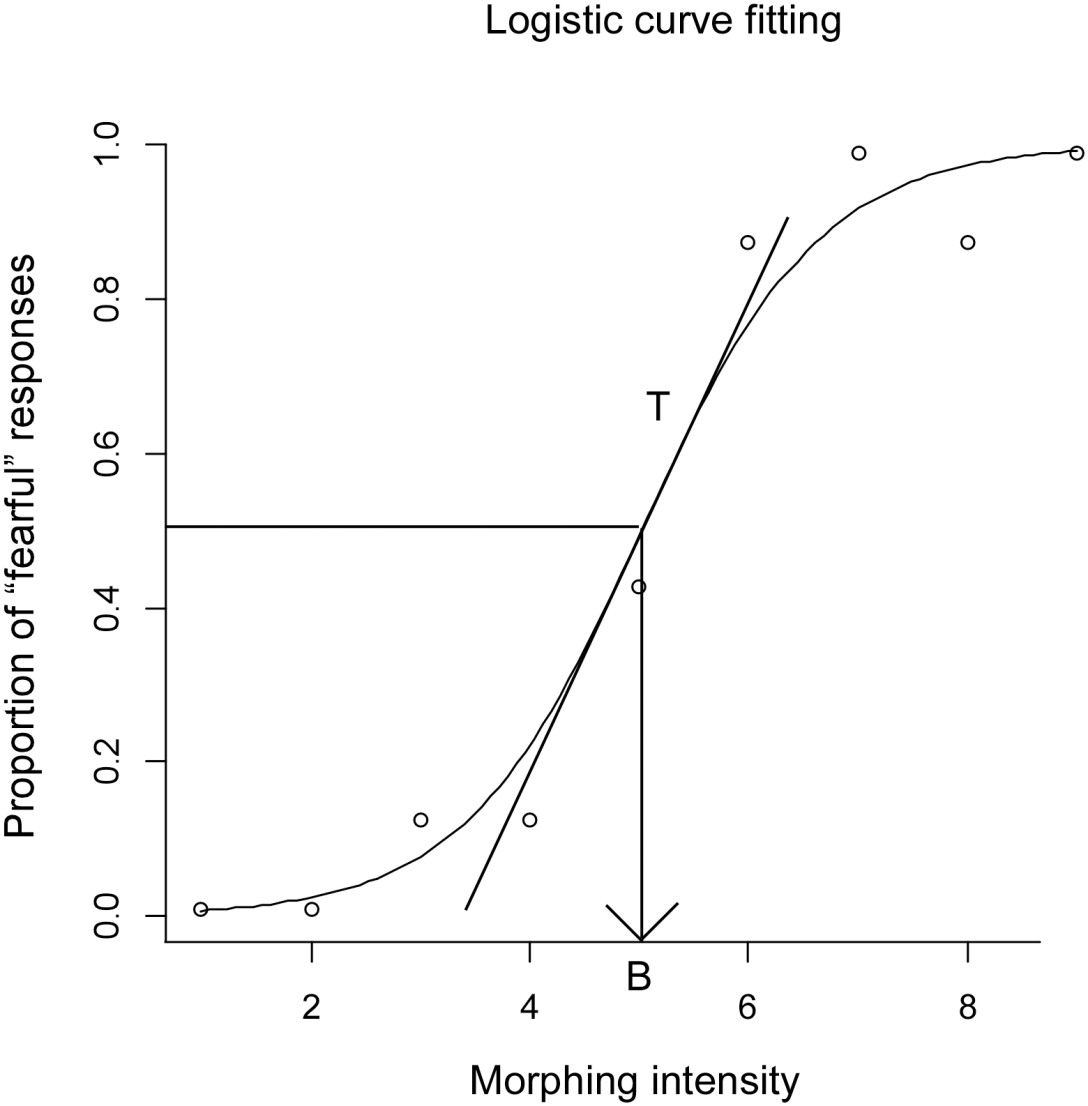
Example of the results from the identification task, fit to a logistic curve. The *x*-axis represents morphing intensity (from face-1 to face-9). The *y*-axis indicates the proportion of trials in which the participant identified the target face as fearful. The category boundary was estimated by the *x*-coordinate at the point on the logistic curve where the *y*-coordinate was 0.5 (“B” on the *x*-axis). The slope of a tangential line at the category boundary (line “T”), which corresponds to 1/4 of b in the logistic regression equation, is considered to be an estimate of categorization strength.

Fig 3 shows the result of the identification task for one condition from a representative subject. The *x* axis indicates the morphing intensity of the target face (1–9), and the *y* axis indicates the proportion of trials in which the participant identified the target face as fearful. The relationship between *x* and *y* is described as a sigmoidal function (Eq 1) using a standard data fitting method, the generalized linear model (GLM) [40].

$$y=\frac{1}{1+e^{-\left(a+bx\right)}}$$ (1)

To avoid function divergence in the GLM, *y* = 0 and *y* = 1 were treated as *y* = 0.01 and *y* = 0.99, respectively. In the current study, we use two indexes to evaluate CP based on the result of the identification task: (1) the inflection point of the logistic curve and (2) the slope of a tangential line. The inflection point, that is, the *x*-coordinate of the point on the logistic curve where the *y*-coordinate was 0.5 [14], can be regarded as the “category boundary” of happy and fearful expressions [20]. The slope of a tangential line at the category boundary can be considered as the extent to which ambiguous faces are categorized as distinct category. Thus the slope of a tangential line can be considered an index indicating “strength” of CP [20]. Logistic model fitting was conducted for each condition for all participants. The range of R-square, which indicates the goodness of fit, was .536–.960 (mean = .820 and SD = .0872).

**Fig 4:**
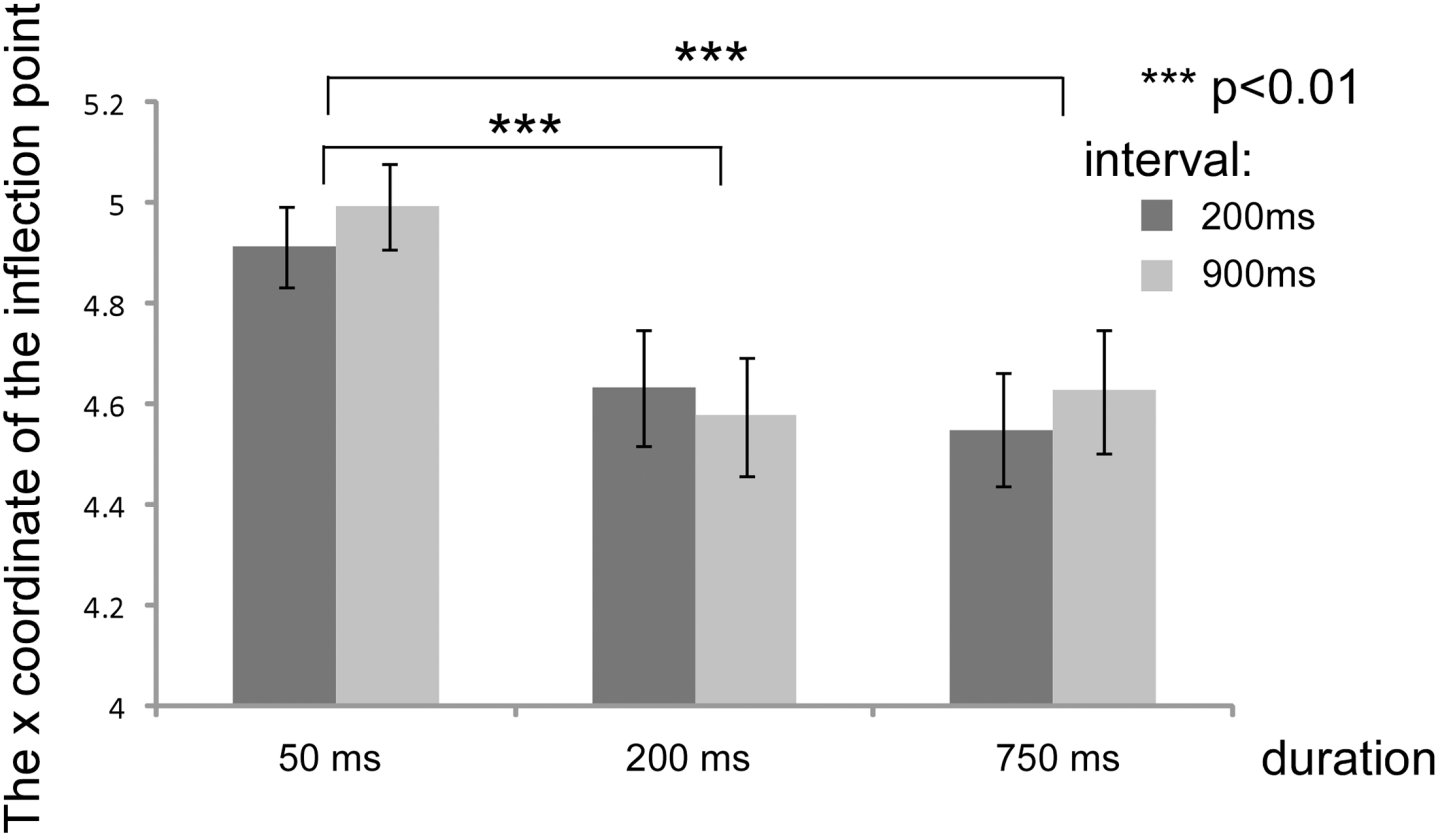
Changes in the category boundary in the identification task. The *y*-axis represents the mean of the morphing intensity of the category boundary. The differences between durations of 50 and 200 ms and between 50 and 750 ms are significant. Error bars indicate the standard error.

Fig 4 shows the average category boundary from all participants for each experimental condition. A two-factor within-subject ANOVA (duration × delay) revealed a significant main effect of duration (*F*(2, 40) = 9.02, *p* < .001, η^2^ = .110). There was no significant main effect of delay, nor a significant interaction. Multiple comparisons (Tukey’s HSD) revealed that the category boundary (i.e., inflexion point of the logistic curve) was larger at 50 ms than at 200 and 750 ms (HSD = 0.265, α = .05), meaning that the estimated category boundary shifted toward “happier” facial expressions at longer durations.

**Fig 5:**
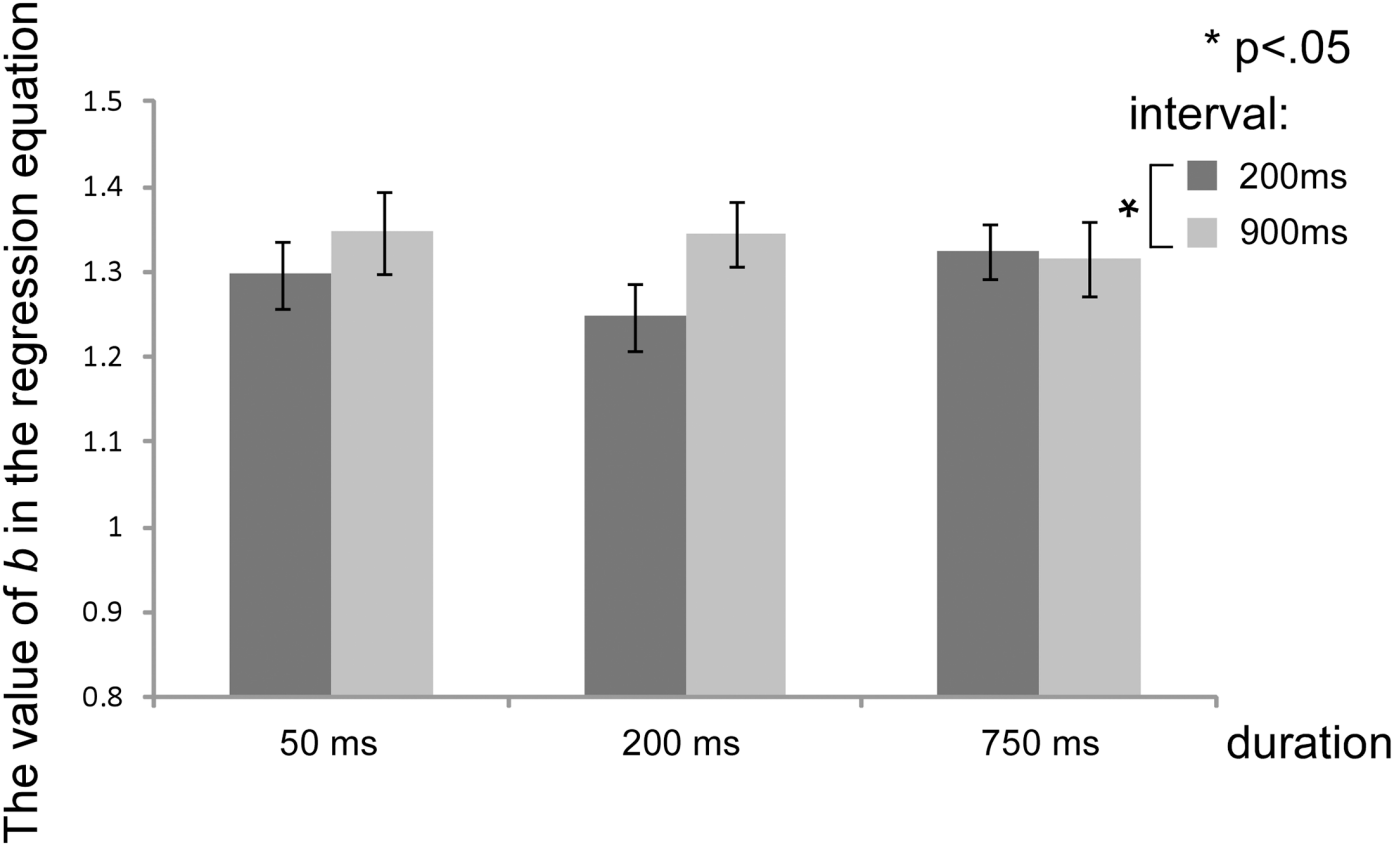
Changes in the strength of CP in the identification task. The *y*-axis indicates the mean of the b parameter estimate in each logistic regression equation. There are significant differences between the 50 and 750 ms delays. Error bars indicate the standard error.

Fig 5 shows the average b parameter, which corresponds to four times the slope of the tangential line at the category boundary, for all participants for each experimental condition. A two-factor within-subject ANOVA revealed no significant interaction, and a significant main effect of delay (*F*(1,20) = 5.71, *p* < .05, η^2^ = .015), such that the slope was smaller in the 200 compared to the 900 ms delay condition. There was no significant main effect of duration.

The raw data of the current task are available in S1 Dataset (Compressed ZIP file.)

### 3.2. Discrimination task

**Fig 6:**
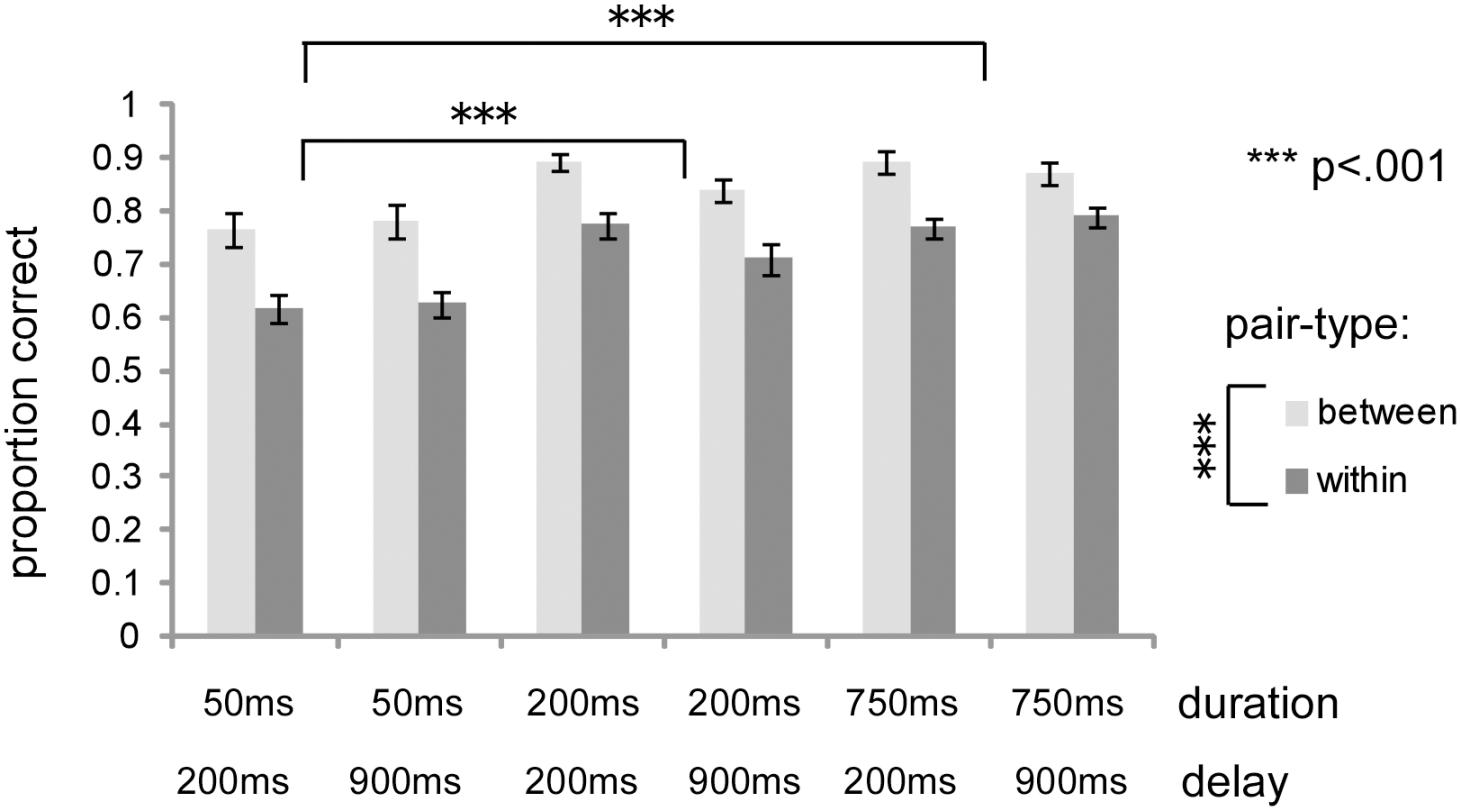
Proportion of correct responses for between- and within-category pairs in the discrimination task. The *y*-axis shows proportion correct. “Between” indicates proportion correct for between-category pairs, while “within” represents the proportion correct for within-category pairs.

Fig 6 shows the proportion of correct responses in each condition in the discrimination task. If perception is categorical, pairs of faces from same category will be less distinguishable than faces from different categories [41]. Therefore, the difference in the proportion of correct responses between between-category and within-category pairs indexes CP. A three-factor within-subject ANOVA revealed a marginally significant interaction between duration and delay (*F*(2,40) = 2.69, *p* < .10, η^2^ = .012), and significant main effects of pair-type (*F*(1,20) = 75.09, *p* < .001, η^2^ = .208) and duration (*F*(2,40) = 24.98, *p* < .001, η^2^ = .170). On the main effect of duration, multiple comparisons (Tukey’s HSD) revealed that proportion correct was significantly lower in the 50 ms duration condition than in the 200 and 750 ms conditions (HSD = 0.076, α = .05).

Based on the results of the identification task for all experimental conditions (see Fig 4), category boundary was determined to be between face-4 and face-5. The face-3/face-5 and face-4/face-6 pairs are between-category pairs, whereas the face-1/face-3 and face-7/face-9 pairs are within-category pairs. This pair-type condition was included in a 3 (duration) × 2 (delay) × 2 (pair-type) ANOVA on proportion correct.

To assess whether CP occurred, we applied a hypothetical method for estimating CP [11, 12]. In this method, discrimination between two facial expressions is assumed to depend on two cues: the physical difference between stimuli and the emotional categories to which the stimuli belong. To estimate the contribution of physical difference, the mean of the observed correct response rate at the end of each continuum (i.e. faces 1–3 and faces 7–9) was used, where the CP effect contribution was the smallest [11]. To estimate the contribution of emotional category, the difference between the identification rates for the two relevant stimuli were multiplied by 0.25. The theoretical correct rate is the sum of the first and second estimates. The constant 0.25 was arbitrary, that is, it has no effect to the correlation coefficient [11]. Therefore, the theoretical correct rate stems from both contributions of the physical and categorical difference. The correlation between the observed data and this theoretical prediction for each facial expression pair in the discrimination task can be used to index CP. In all conditions, the correlation was significant (50 ms duration/200 ms delay condition: *r* = .844, *t*(5) = 3.524; 50 ms duration/900 ms delay condition: *r* = .812, *t*(5) = 3.114; 200 ms duration/200 ms delay condition: *r* = .872, *t*(5) = 3.982; 200 ms duration/900 ms delay condition: *r* = .875, *t*(5) = 2.834; 750 ms duration/200 ms delay condition: *r* = .958, *t*(5) = 7.458; 750 ms duration/900 ms delay condition: *r* = .869, *t*(5) = 3.928; all *p*s < .05). S1 Fig shows the observed performance and theoretical performance at each level of morphing in each condition.

The raw data of the current task are available in S1 Dataset (Compressed ZIP file.)

In order to investigate if the CP modulation in the current study was affected by the stimulus repetition and the priming, we conducted the analysis of the former part (early 1/3 trials) of each block and the analysis of the priming effect. The analysis using former part of the block showed the main effect of duration, interval and current pair, and the analysis using all trials showed the interaction of the priming and current pair. The additional analysis using all trials showed that the extent of CP was higher if the within-category pair preceded (see S2 Fig). On the other hand, the priming effect was not observed in the analysis using former part of the block (for the more detailed result, see S1 Text).

## Discussion

In the current study, the contribution of temporal factors to CP of facial expression was investigated by manipulating temporal parameters of the tasks. Although the sampling of temporal factors may not be enough to allow a parametric analysis, the current study marks important progress in advancing the study for CP of facial expression from the temporal aspect.

The results from the discrimination task demonstrated that, for all duration and delay conditions, proportion correct was significantly higher for between-category compared to within-category pairs. This suggests that CP occurs even when the stimulus duration is 50-ms and the delay is 200-ms. This is also supported by the analysis of the theoretical CP estimates. That is, theoretical predictions and the experimental observations were highly correlated (*r*s > .8, *p* < .05) for all duration and delay conditions, indicating the occurrence of CP [11, 39]. These results are consistent with previous studies suggesting that CP occurs even at the early stages of facial expression recognition processing [21].

Furthermore, the current study showed that, in the identification task, the slope at the inflexion point changed according to delay length; that is, when the delay was long, the slope was steep. The slope at the inflexion point can be considered the extent to which ambiguous faces are categorized as distinct category. This suggests that, even though the stimulus presentation duration was the same, the strength of CP increased according to delay length. Some may argue that this change in performance is due to differences in task difficulty. In fact, the low performance in the 50-ms duration conditions may reflect task difficulty. However, the observed changes with stimulus delay cannot be explained by task difficulty. In general, tasks requiring memory should be more difficult when delays are longer [34]. In addition, if a participant did not know the answer in the identification task and had to guess, the resulting slope of the logistic curve would be more gradual. However, in the current experiment, the change in slope as a function of delay was antipodal to the effect expected based on task difficulty. Therefore, the steeper slope with longer delays is not thought to be due to task difficulty.

Given the above discussion, this change in slope may reflect internal processing assumed to occur during the delay period. Such internal processing may include sensory-retention in lower sensory levels of processing, and verbalization for memory as part of higher cognitive functions. We hypothesize that higher cognitive functions, such as verbal labeling, may be more responsible for the observed changes in CP strength, i.e. in the long delay condition, participants may use verbal labeling of the stimuli to memorize them resulting in higher CP strength. This is consistent with recent studies showing that verbal interference attenuates CP of facial expression [15]. The current result is compatible with the two-pathway theory, in that the slower pathway activates in longer delay condition, and the participants can take more deliberate judgment.

Some researchers suggest that the current results are inconsistent with a previous study showing that CP is not observed if target stimulus presentation duration is very short (75 ms) [20]. This inconsistency could be due to a floor effect. In the current study, the gap between paired faces was 25%, whereas in Experiment 2 of [20] the gap was 20%, resulting in a more difficult task. Additionally, the task is more difficult when stimulus duration is short. Therefore, task difficulty may reduce correct response rate for between-category pairs.

We also observed that the estimated category boundary (i.e., inflexion point of the logistic curve) shifted toward “happier” facial expressions with longer durations. That is, stimuli were identified as more fearful when the stimuli were presented for longer durations. This is consistent with previous studies showing that more time is required to process faces expressing fear [42, 43, 44, 45]. Research also suggests that neutral faces are perceived as more negative when they are presented longer [30]. The current experiment supports those observations in the context of CP.

Remarkably, a shift in category boundary was observed only with changes in stimulus duration, not changes in delay. This dissociation between the impact of stimulus duration and delay suggests that the shift in category boundary associated with stimulus duration may reflect a different process, such as stimulus accessibility. For example, in visual search tasks, happy faces can be detected faster than faces expressing other emotions [46], meaning that happy faces can be detected with fewer resources. This is consistent with the current results in the sense that faces tended to be perceived as happy with shorter presentation durations (with fewer resources), whereas faces tended to be perceived as fearful with longer presentation durations (with greater resources). These findings may seem to be inconsistent with the concept of “negativity bias” [47], which claims that negative stimuli stand out and attract more attention, because the rapid detection of fearful stimuli is considered to be adaptive. However, previous studies suggested that in categorization task, it requires more time to make a cognitive judgment on fearful stimuli because the urgent message (“danger!” etc.) may act as the distractor that inhibits categorization [45]. Therefore the current results are also consistent with the concept of negativity bias in the sense that, when the stimulus duration increases, the accessibility of the stimulus may increase, and therefore negative stimuli may stand out more.

One may argue that the manipulation of delay cannot make an impact to the retention of the stimuli, because the answer was presented until participants responded. However, the RT of each delay condition (200 ms and 900 ms) was respectively 892ms and 978ms, the difference between which is not so far different as the difference of delay condition (200 ms and 900 ms) is. Therefore, we concluded that the impact of delay to the retention exists.

One may argue that attention should be paid to the interpretation of the current results from the aspect of stimulus specificity. However, it is less likely that the current results are distorted by the stimulus selection for the following reasons. First, although participants are less sensitive to expression changes if the actors are different race than participants' own race (other-race effect, see [48]), the model identity (a European male) used in the current study has been repeatedly used for testing the CP of facial expression. Such previous studies including Calder's work [11] have provided evidence for CP of facial expressions, even though their studies only used single model identity. Moreover, fearful-happy continuum can be regarded as well-controlled stimuli to test the CP of facial expression in the sense that fearful and happy expressions have same levels of arousal and are located at the opposite poles on the dimension of emotional valence [39]. Second, although Japanese participants have been considered to confuse the fear faces with surprise faces and disgust faces due to the difference in strategy of visual search for facial expressions [49, 50, 51], recent study confirmed that Japanese participant can correctly categorize fearful faces as negative stimuli [39]. Therefore, as long as emotional valence is concerned, the Japanese participants in the current study could identify and discriminate the facial stimuli. An important issue for future research will be to investigate whether the current observations can be generalized across stimulus features such as facial models' sex, age, race, types of facial expression, attractiveness, and familiarity.

In the current research, we used the static images, which are scarcely observed in the real world. Therefore, participants might use a strategy which is useful only for the answering in the current trial, not useful for the real emotional processing. However, many previous research on facial expression used the static images [10, 11, 14, 42, 45], and these researches showed important results. Therefore, we think that using static images is valid, though they are scarcely observed in the real world. Further study using dynamic images will be informative for where and when processing of emotional stimuli occurs in the real information processing.

In the current study, we used the limited number of stimuli and these stimuli are presented repetitively. This is inevitable with the respect to the experimental design in order to obtain stable data of the proportion correct and to test the 6 conditions with the different temporal parameters. However, with respect to the repetitive presentation of the stimuli, we should consider the possibility that the saturation/habituation of the stimuli may affect the result. In addition, we should also consider the possibility that the result of a trial could be affected by its preceded trial (priming effect). For example, a recent study showed that the type of stimulus of the previous trial affects the extent of CP in the next trial [52].

In order to test these possibilities, we conducted the additional analysis. In the analysis using the former part (1/3 of all trials) of the block, we observed the main effects of the duration, the delay and the current pair. This result suggests that the saturation of the stimuli has little effect to the change of the CP extent induced by the temporal factors. In the analysis of the priming effect, using all trials of the block, we observed that the extent of CP was higher when the within-category pair preceded. However, we observed neither the main effect of priming nor the simple main effect of priming, and therefore the priming effect did not affect the CP modulation by temporal factors.

## Supporting Information

S1 Dataset Raw data of identification task and discrimination task. (ZIP)

S1 Fig The observed and theoretical proportion correct at each level of morphing in each condition. (TIF)

S2 Fig The interaction between priming pair and current pair. The proportion correct of the between-category pair is higher when the within-category pair is preceded, whereas the proportion correct of the within-category pair is higher when the between-category pair is preceded.
(TIF)

S1 Text Supplementary Analysis. (DOC)
