# Supplementary figures and images for: Modulation of Emotional Category Induced by Temporal Factors in Emotion Recognition
Source: PLoS One. 2015 Jul 31;10(7):e0131636. doi: 10.1371/journal.pone.0131636 (PMC4521787; doi:10.1371/journal.pone.0131636)

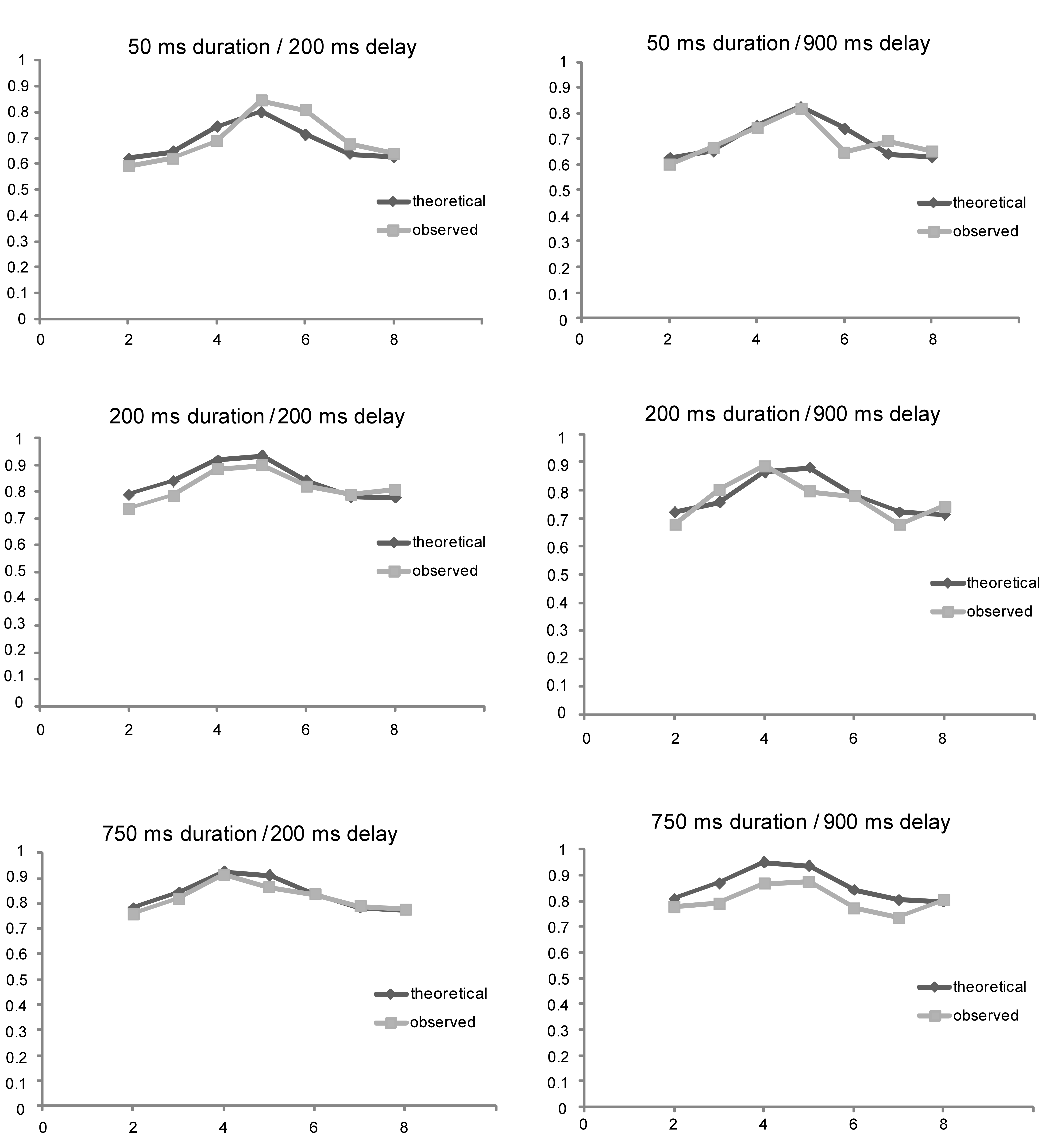

Supplement: S1 Fig — (TIF) [file pone.0131636.s002.tif]

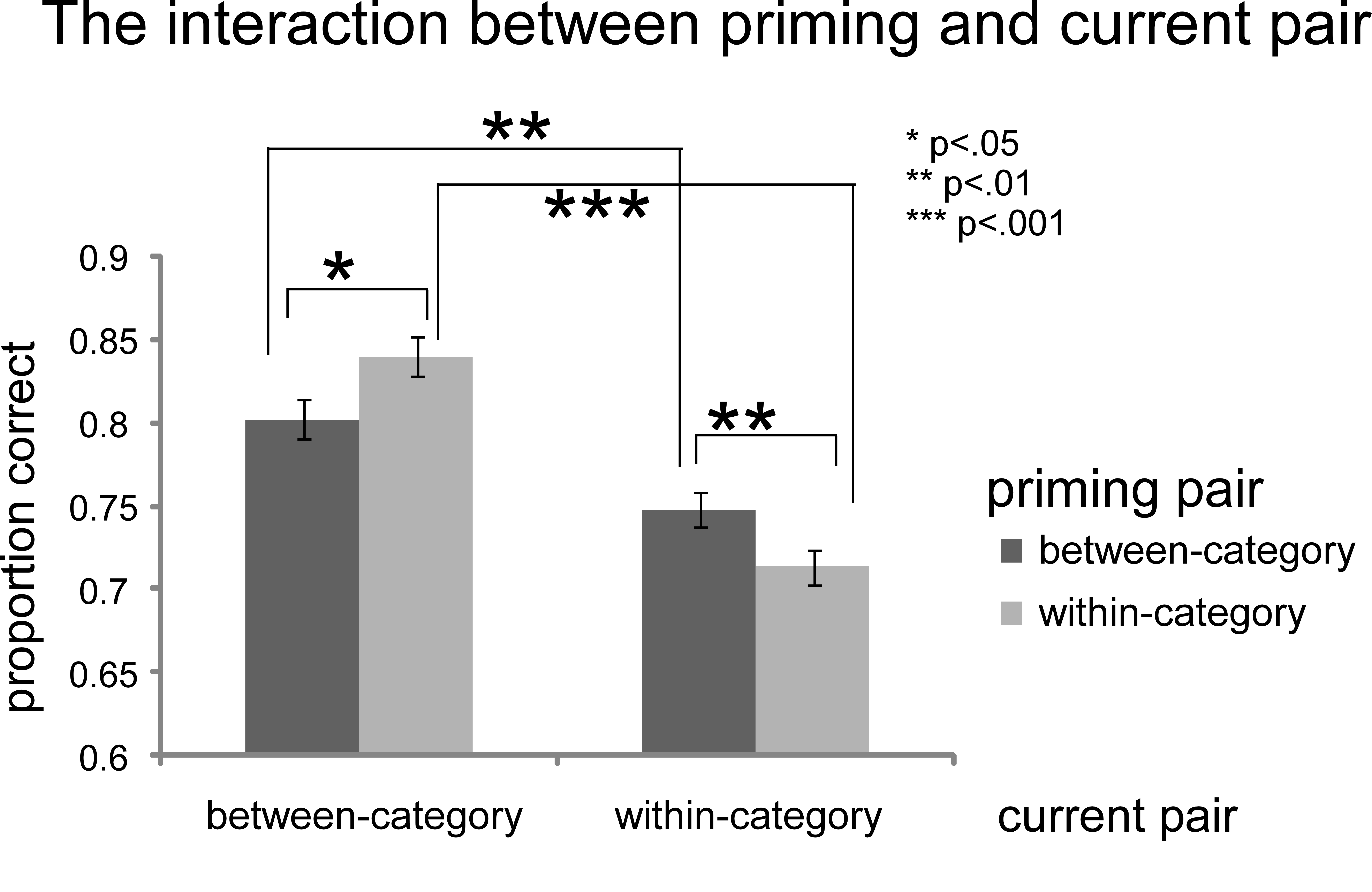

Supplement: S2 Fig — The proportion correct of the between-category pair is higher when the within-category pair is preceded, whereas the proportion correct of the within-category pair is higher when the between-category pair is preceded. (TIF) [file pone.0131636.s003.tif]
