# Supplementary material for: Modulation of Emotional Category Induced by Temporal Factors in Emotion Recognition
Source: PLoS One. 2015 Jul 31;10(7):e0131636. doi: 10.1371/journal.pone.0131636 (PMC4521787; doi:10.1371/journal.pone.0131636)
Supplement: S1 Text — (DOC) [file pone.0131636.s004.doc]

**Supplementary analysis**

In the current research, we investigated the modulation of the CP extent by manipulating the stimulus duration and delay. However, the recent research shows that the pair-type (between-category pair or within-category pair) of the previous trial affects the extent of CP in the next trial [1]. In order to investigate if the CP modulation in the current study resulted from the effect of the previous trial, we additionally analyzed the effect of the previous trial in the discrimination task.

We added the “priming pair” factor to each trial. If the pair used in previous trial was “between-category” pair, the “priming pair” was “between-category”, irrelevant to the pair in the current trial. Afterward, we conducted ANOVA for the proportion correct.

The four-way (duration, delay, priming pair, current pair) ANOVA revealed that the significant main effects of duration (*F*(2,40) = 35.99, *p*<.001), delay (*F*(1,20) = 19.01, *p*<.001) and the current pair (*F*(1,20) = 61.83, *p*<.001). The analysis also revealed the significant interaction of duration and priming pair (*F*(2,40) = 3.953, *p*<.05), and the significant interaction of priming pair and current pair (*F*(1,20) = 13.59, *p*<.01), marginally significant interaction of duration and current pair (*F*(2,40) = 3.039, *p*<.10). Figure S2 shows the proportion correct with the condition of priming pair and the current pair.

In the current analysis, we used all pairs for the analysis, namely, the pairs between face 3 and face 5, face 4 and face 6, and face 5 and face 7 are regarded as between-category pairs, and the rest are regarded as within-category pairs.

*The analysis of interaction between priming and duration*

In the analysis of duration and priming pair interaction, multiple comparison revealed that the simple main effect of duration. The Tukey’s HSD revealed that in the between-pair priming condition, the proportion correct was significantly different (*p*<.001) in 50ms and 200ms condition and 50ms and 750ms condition. Moreover, in the within-pair priming condition, the proportion correct was significantly different (*p*<.001) in 50ms and 200ms condition and 50ms and 750ms condition.

*The analysis of interaction between priming and current pair*

Multiple comparison revealed that the simple main effect of priming pair and current pair. In between-pair priming condition and within-pair priming condition, the proportion correct of between-pair was significantly higher than that of within-pair (*F*(1,20) = 10.14, *p*<.01; *F*(1,20) = 98.38, *p*<.001). Moreover, In between-pair condition of the current pair, the proportion correct was significantly higher in within-pair priming than in between-pair priming (*F*(1,20) = 5.524, *p*<.05), and in within-category condition of the current pair, the proportion correct was significantly lower in within-pair priming than in between-pair priming (*F*(1,20) = 8.674, *p*<.01).

*The analysis of saturation*

In addition to these results, we conducted the same analysis using the former trials (1/3 of all trials) of the block. The four-way ANOVA revealed that the main effect of duration (*F*(2,40) = 4.746, *p*<.05), delay (*F*(1,20) = 9.82, *p*<.01) and current pair(*F*(1,20) = 26.11, *p*<.001), but there was neither significant main effect of priming pair nor the interaction between priming pair and any other factors.

**Discussion**

In this additional analysis, we observed that there is no significant main effect of priming, and in the analysis of interaction between duration and priming, there is no significant simple main effect of priming. Therefore, we conclude that in the current study, the CP modulation effect of duration and delay is irrelevant to the priming effect.

In addition, the result of additional analysis indicated that the proportion correct of between-category pair is higher than that of within-category pair in both between-category pair priming condition and within-category pair priming condition. This suggests that, contrary to the previous research [1], the priming effect on the extent to CP was not observed in the current research.

*The label-conflict hypothesis*

A recent research shows that in the discrimination task, the extent of CP is higher when the stimulus of the previous trial is the between-category pair than the within-category pair [1]. They try to explain this effect of priming based on the category label comparison model. According to the label comparison model, CP (advantages in discrimination in between-category pair than in within-category pair) occurs by the conflict between verbal label and perceptual label. The model suggests that the between-category pair is more distinguishable than the within-category pair because there is no conflict between verbal label and perceptual label.

Based on this model, they [1] claimed that participants give weight to the categorical code (verbal label) after the between-category pair priming, whereas the influence of categorical code is reduced by the label-conflict of the within-category pair priming. These explanations justified the result of the previous experiment [1], in which the strong CP effect emerged after between-category pair priming.

However, this additional analysis demonstrated that the effects of priming pair are different from the results of Hu (2014). First, the difference of proportion correct between within-pair and between-pair was significant in both between-pair priming and within-pair priming condition. Second, in the previous research, the RT was faster when the priming pair and the current pair were congruent (say, within-priming pair and within-current pair) than when they were incongruent (say, between-priming pair and within-current pair), whereas in the current research, the proportion correct was higher when the priming pair and the current pair was incongruent than when they were congruent. One possible explanation for this observation is that the examples of one category activate the difference between the categories [2]. For example, the “fearful” within-category pair priming activates the “fearful” category label. Therefore, when the between-category pair is presented after within-category pair, the perceived difference may be large, and the proportion correct becomes higher.

On the other hand, when within-category pair is presented after within-category pair, the corresponding category is activated and the difference between the examples from the same category (within-category pair) is ignored, resulting in the lower the proportion of correct responses.

Moreover, in the current research, the overall proportion correct was lower than that of the previous research [1]. Therefore, the perceptual label is not necessarily different in the within-category pair discrimination, and the conflict of perceptual/verbal labels is weak. In sum, the label-conflict hypothesis is not compatible with the current experiment. This is perhaps the reason why the results of the current research and the previous research [1] are different.

In addition, the interaction of priming was not observed in the analysis using the former trials of the block. This suggests that the priming effect is observed only after the habituation of the stimuli.

*The effect of saturation*

In the analysis using the former part (1/3 of all trials) of the block, we observed the main effects of the three factors (the duration, the delay and the current pair). This result suggests that the saturation of the stimuli has little effect to the change of the CP extent induced by the duration or delay. In the analysis of the priming effect, using all trials of the block, we observed that the CP extent was higher when the within-category pair preceded. However, we observed neither the main effect of priming nor the simple main effect of priming, and therefore the priming effect did not affect the CP modulation by temporal factors. On the other hand, In the analysis using the former part of the block, we observed neither the main effect of priming nor the interaction between priming and other factors. This suggests that the priming effect in the current research occurs only when participants are habituated the stimuli. These observations suggest that we should pay attention to the priming effect when we use the experimental paradigm in which the limited numbers of stimuli were presented repetitively [1].

References

[1] Hu Z, Hanley JR, Zhang R, Liu Q, Roberson D (2014) A conflict-based model of color categorical perception: evidence from a priming study. Psychon Bull Rev 21: 1214-1223. doi: 10.3758/s13423-014-0603-8

[2] Lupyan G (2012) Linguistically modulated perception and cognition: the label-feedback hypothesis. Front Psychol 3:54. doi: 10.3389/fpsyg.2012.00054
